# Isolation of Red Beet Plant-Derived Nanovesicles, and Characterization of Their Molecular Content and Biological Activities in Human Cells

**DOI:** 10.3390/ijms262311261

**Published:** 2025-11-21

**Authors:** Clarissa Zanotti, Antonio Dario Troise, Simona Arena, Giovanni Renzone, Sabrina De Pascale, Rosalia Ferracane, Chiara Pontecorvi, Chiara Niespolo, Angelo Gismondi, Andrea Scaloni, Mauro Marra

**Affiliations:** 1 Department of Biology, University of Rome “Tor Vergata”, Via della Ricerca Scientifica 1, 00133 Rome, Italy; zanotti.clarissa97@gmail.com (C.Z.); chiara.pontecorvi@uniroma2.it (C.P.); gismondi@scienze.uniroma2.it (A.G.); 2 PhD Program in Cellular and Molecular Biology, Department of Biology, University of Rome “Tor Vergata”, Via della Ricerca Scientifica 1, 00133 Rome, Italy; 3 Proteomics, Metabolomics and Mass Spectrometry Laboratory, ISPAAM, National Research Council, P.le E. Fermi 1, 80055 Portici, Italy; antoniodario.troise@cnr.it (A.D.T.); simona.arena@cnr.it (S.A.); giovanni.renzone@cnr.it (G.R.); sabrina.depascale@cnr.it (S.D.P.); rosaliaferracane@cnr.it (R.F.); andrea.scaloni@cnr.it (A.S.); 4 Arterra Bioscience SpA, Via Benedetto Brin 69, 80142 Naples, Italy; chiara@arterrabio.it

**Keywords:** plant-derived nanovesicles, *Beta vulgaris* L., proteomics, lipidomics, miRNomics, cross-kingdom regulation, wound healing

## Abstract

Nowadays, growing evidence indicates that plant-derived nanovesicles cross biological barriers between species, including humans, and deliver therapeutic molecules that influence gene expression, affecting various processes such as inflammation, oxidative stress, and cancer. For these reasons, plant-derived nanovesicles are gaining attention as a valuable substitute for mammalian exosomes as they offer benefits such as reduced immunogenicity, enhanced bioavailability, and the inclusion of beneficial plant metabolites. However, the development of affordable plant-derived nanovesicle-based therapies requires a robust characterization of their molecular structure and cargo, which in turn depends on obtaining sufficient quantities of homogeneous nanovesicle populations. In this study, we used an advanced purification platform combining ultrafiltration and anion exchange chromatography to isolate highly pure plant-derived nanovesicles from a new source, *Beta vulgaris* L. These particles were characterized in terms of size, charge, and morphology, and their molecular content was analyzed by omic technologies, including proteomics, lipidomics, and miRNomics. Their ability to promote wound healing and reduce inflammation was demonstrated in vitro using human cells. Furthermore, bioinformatic analysis linking the microRNA profile with potential human target genes provides insights into the biochemical pathways that underlie the bioactivity of nanovesicles.

## 1. Introduction

Exosomes (20–200 nm) are the smallest fraction of extracellular vesicles, nanoparticles ranging from 20 to 1000 nm in diameter produced by cells from various organisms, including animals and plants. Mammal-derived exosomes (MDEs) are secreted into extracellular fluids, travel to, and are internalized into target cells, where they release a wide variety of bioactive molecules, including proteins, lipids, nucleic acids, and metabolites [1,2]. These molecules can influence gene expression as well as physiological and pathological processes in host cells [3,4]. Human exosomes (HEs) have been extensively studied due to their potential therapeutic applications in the treatment of various diseases [5,6,7,8].

Recently, plant-derived nanovesicles (PDNVs) have attracted research interest, prompted by observations that consuming certain foods is linked to a lower risk of various diseases, including inflammatory bowel disease, diabetes, hypertension, ischemia, and cancer [9,10,11]. PDNVs from edible plants can be absorbed via the gastrointestinal tract [12,13] and transport bioactive molecules such as miRNAs, proteins, lipids, and secondary metabolites [14]. Plant-derived miRNAs have been found to regulate human genes involved in inflammation, oxidative stress, and cancer [9]. The therapeutic application of HEs is limited by several complications, including immunogenicity, cytotoxicity, pathogen contamination, and high production costs. In contrast, PDNVs offer several advantages such as lower immunogenicity, higher bioavailability, absence of human pathogen contamination, and greater availability [15]. Although they differ in some aspects, PDNVs are comparable to HEs regarding their size, structure, and ability to deliver bioactive molecules to target cells. Although numerous studies have demonstrated the therapeutic potential of PDNVs and some clinical trials are currently underway [9], our understanding of their mechanism(s) of interaction with mammalian cells remains limited. To pave the way for more targeted applications of PDNVs, several knowledge gaps must be addressed, including mechanisms of recognition and internalization in target cells, pharmacokinetic properties in human fluids, and the affected molecular pathways. A key prerequisite for elucidating their mechanisms of action is the availability of sufficient quantities of highly purified PDNVs, enabling a robust characterization of their molecular structure and cargo. Currently, PDNVs have been purified by various techniques, none of which fully meet the yield, purity, reproducibility, and scalability requirements. In a previous study, we established a purification method for PDNVs by adapting a procedure originally developed for isolating HEs from cell culture extracellular fluids [16,17]. This method combines ultrafiltration and anion exchange chromatography in an FPLC system, ensuring the reproducible isolation of PDNVs from plant tissue extracts with high yield and homogeneity [18].

In this study, we applied the above-reported method to purify PDNVs from beetroot (*Beta vulgaris* L.) seedlings. *B. vulgaris* is a Mediterranean species of the Chenopodiaceae family, which has been used since ancient times in traditional medicine. It contains specific classes of bioactive secondary metabolites such as betalains in addition to flavonoids and polyphenols, and previous studies have demonstrated that it possesses antioxidant, anticancer, hepatoprotective, nephroprotective, wound-healing, and anti-inflammatory activities [19].

PDNVs with a high purity index have been obtained and characterized in terms of dimension, surface charge, and morphology using nanoparticle tracking analysis (NTA) and transmission electron microscopy (TEM). Their molecular content has been further characterized by proteomic, lipidomic, and miRNomic analyses. Additionally, their wound-healing and anti-inflammatory effects have been assessed in vitro using human cell cultures. Bioinformatics was then used to correlate these biological activities with the ability of specific miRNA cargo species to modulate the transcription of potential human target genes [11,17,20].

## 2. Results

### 2.1. Purification by B. vulgaris PDNVs by Anion Exchange Chromatography

**Figure 1:**
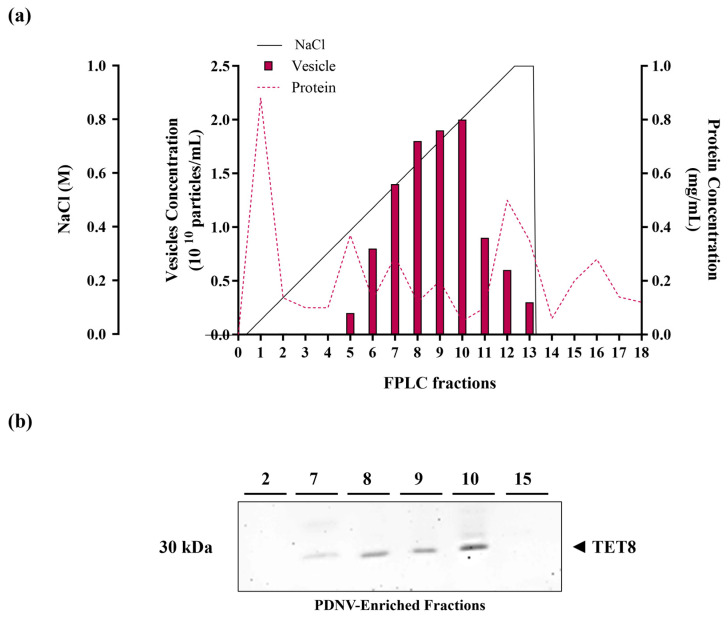
Anion exchange chromatography purification of *B. vulgaris* PDNVs. (**a**) FPLC chromatogram displaying the elution profiles of proteins and PDNVs. The ultrafiltered sample was applied to a CIMmultus™ EV anion exchange monolith column and eluted using a 0–1 M NaCl linear gradient, as reported in the Materials and Methods. (**b**) Western blot analysis of the eluted fractions. Samples from fractions 2, 7–10, and 15 were analyzed by SDS-PAGE and probed with an anti-TET8 antibody under the conditions reported in the Materials and Methods. A representative experiment is shown.

Following centrifugation to remove large cellular debris, the homogenate obtained from the aerial parts of *B. vulgaris* seedlings underwent sequential filtration, followed by ultrafiltration to remove low-molecular-weight contaminants. The resulting sample was then subjected to diafiltration to enable buffer exchange prior to nuclease treatment to remove high-molecular-weight aggregates of nucleic acids and proteins. After nuclease treatment, the sample was diafiltered again using an anion exchange buffer and then further purified via anion exchange chromatography on an FPLC system. The elution was carried out with a linear 0–1 M NaCl gradient for 35 min. Protein concentrations in the collected fractions were determined using the Bradford assay, while PDNV concentrations were measured by NTA analysis. The elution profile (Figure 1, panel a) indicated that the PDNVs were concentrated in fractions 7–10, whereas most contaminating proteins were eluted at either lower or higher NaCl concentrations. To confirm the presence of PDNVs, these fractions were analyzed by Western blotting using antibodies against TET8, a well-established PDNV marker. A strong signal was observed in fractions 7–10, corresponding to the PDNV-containing fractions, while no signal was observed in other fractions, such as fractions 2 and 15, which contained most of the contaminant proteins (Figure 1, panel b).

**Figure 2:**
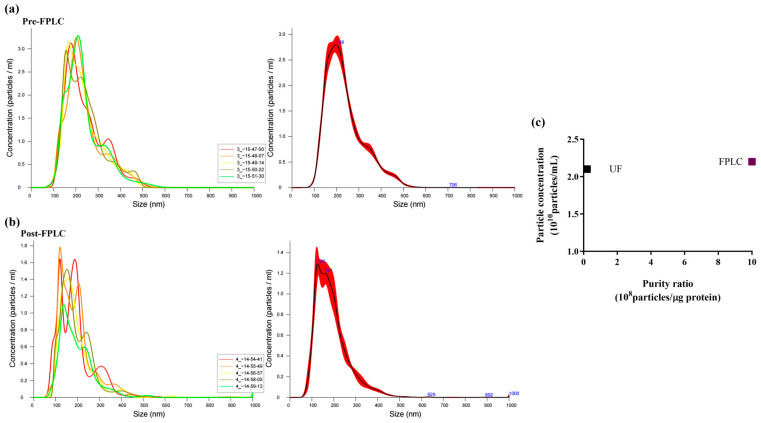
Nanoparticle Tracking Analysis (NTA) of the ultrafiltered (pre-FPLC) *B. vulgaris* sample and the FPLC-purified pooled 7–10 fractions (post-FPLC). (**a**) NTA-measured size distribution of PDNVs in the pre-FPLC sample. (**b**) NTA-measured size distribution of PDNVs in the post-FPLC sample. (**c**) Purity index, calculated as number of particles/μg protein ratio, for the pre-FPLC sample and the post-FPLC sample, respectively. A representative experiment is shown.

To quantitatively assess the efficiency of anion exchange chromatography purification, NTA analysis was performed on pooled fractions 7–10 (post-FPLC) and compared with the ultrafiltered sample (pre-FPLC). NTA of the pre-FPLC sample (Figure 2, panel a) showed a Gaussian size distribution of PDNVs, with D10 (10% of the population) at 145.5 nm, D90 at 361.6 nm, and an average size of 236.6 nm. On the contrary, the post-FPLC sample (Figure 2, panel b) showed a narrower size distribution, with D10 and D90 values of 71 and 269 nm, respectively, and an average diameter of 155 nm. This analysis demonstrates that the post-FPLC sample contained a more uniform PDNV population due to a significant reduction in high-molecular-weight contaminants, such as protein aggregates and nucleosomes [16]. PDNV concentration was 2.30–2.68 × 10^10^ particles mL^−1^ in the pre-FPLC sample and 1.88–2.10 × 10^10^ particles mL^−1^ in the post-FPLC sample. The purity index, expressed as the number of particles per µg of protein, improved from 0.1 × 10^8^ (pre-FPLC) to 1.0 × 10^9^ (post-FPLC) (Figure 2, panel c). Overall, our results indicate that the anion exchange purification step eliminated approximately 90% of contaminants without significantly affecting the PDNV yield.

**Figure 3:**
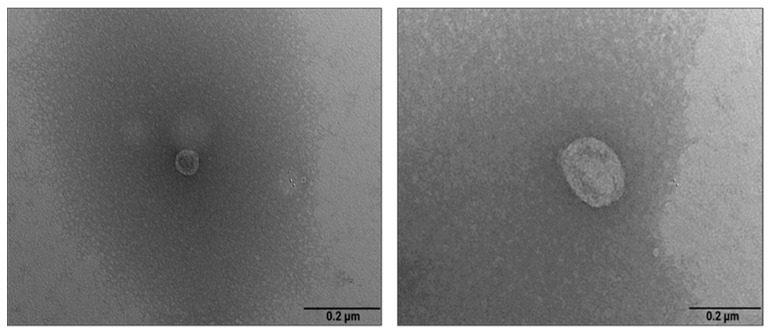
Transmission electron microscopy images of anion exchange-purified *B.* vulgaris PDNVs. The samples were negatively stained, air dried, and analyzed as described in the Materials and Methods. Scale bar  =  200 nm.

Finally, FPLC-purified PDNVs were examined by TEM. As shown in Figure 3, the nanovesicles exhibited a spherical morphology, with a distinct lipid bilayer clearly separated from the surrounding medium. Their size ranged from 20 nm (panel a) to 170 nm (panel b), with an average diameter of approximately 82 nm. The size distribution estimated by TEM analysis was smaller than that obtained through NTA analysis. This discrepancy is likely due to the intrinsic differences between the two techniques, with TEM measuring the physical diameter and NTA evaluating the hydrodynamic diameter of nanoparticles. Additionally, sample preparation artifacts in TEM can cause particle dehydration and collapse, resulting in the reduction in dimensions and cup-shaped nanovesicles. Nonetheless, both analyses confirmed the reliability of the isolation protocol in producing nanovesicles with a narrow size distribution while maintaining their native morphology.

### 2.2. Proteomic Analysis

**Figure 4:**
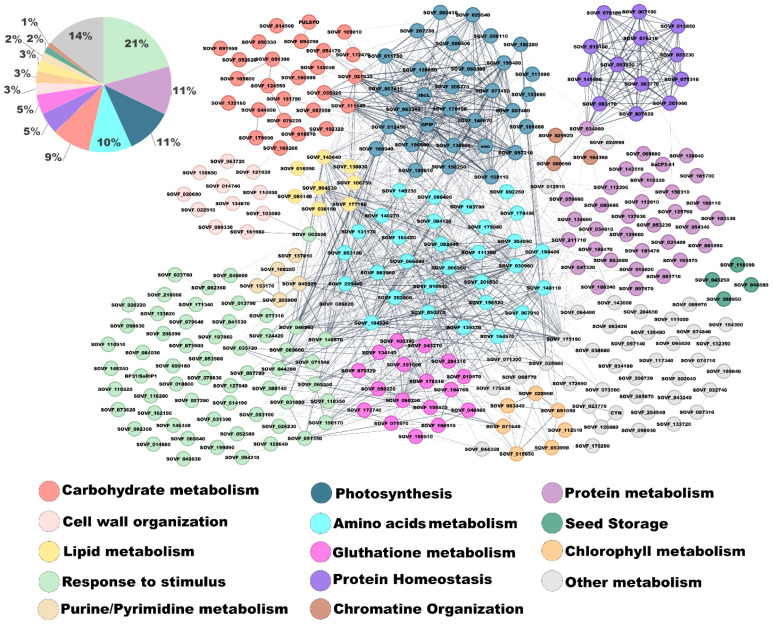
Functional classification and interaction network of proteins identified in *B. vulgaris* PDNVs. The network diagram illustrates the predicted interactions among proteins, where each node represents a gene and each edge indicates a functional or physical association. Nodes are color-coded according to their assigned biological processes, including stress response, metabolism, protein homeostasis, and other cellular functions. The accompanying pie chart summarizes the relative representation of proteins within each functional category.

Overall, 321 proteins were identified in *B. vulgaris* PDNVs through nanoLC-ESI-Q-Orbitrap-MS/MS analysis, combined with a bioinformatic search of the mass spectrometry data against a plant-specific sequence database. Functional annotation of identified proteins and molecular network inference analysis were performed using STRING software v. 12.0 with a confidence score cutoff value of 0.4. Protein annotation was performed using *Spinacia oleracea* homologs because *Beta vulgaris* is still incompletely annotated in current databases and, accordingly, is not reported in the STRING database. Both plant species belong to the same subfamily (Chenopodioideae), and their close phylogenetic relationship supports reliable functional inference. This database choice, commonly applied in proteomic studies of non-model plants, introduced minor uncertainties that are acknowledged as a study limitation. This approach allowed for the recognition of 304 proteins within the *S. oleracea* database and the functional classification of 216 unique genes, which were grouped into 14 biological process categories (Figure 4), highlighting the complexity and functional diversity of the components occurring in PDNVs. The complete list of identified protein species is reported in Table S1.

The most abundant category was associated with response to stimulus function, comprising 52 genes (24.1% of the total). This group included several oxidative stress-related enzymes, such as peroxidases (SOVF_065840, SOVF_073620, SOVF_092350, SOVF_128640, SOVF_133820, SOVF_146350, SOVF_162150), catalases (SOVF_051350, SOVF_031600), and aldo-keto reductases (SOVF_002600, SOVF_026230, SOVF_046600, SOVF_062360, SOVF_127640), suggesting a role for the nanovesicle protein cargo in redox signaling and cellular defense. Additionally, the presence of germin-like proteins (SOVF_020220, SOVF_071540, SOVF_079640, SOVF_084030, SOVF_116280) and purple acid phosphatases (SOVF_013790, SOVF_041530, SOVF_053980, SOVF_078830) supported the involvement of PDNVs in pathogen defense and cell wall remodeling. An enrichment of stress-related proteins has already been observed in PDNVs from *Arabidopsis thaliana* L., where vesicle secretion increases in response to pathogen attack and oxidative stress [21]. Proteins involved in protein homeostasis and proteasome function were also prominently represented. Multiple subunits of the 20S proteasome core complex were identified, including beta subunits (SOVF_003170, SOVF_007620, SOVF_015190, SOVF_035230, SOVF_078180, SOVF_145550) and alpha subunits (SOVF_007100, SOVF_013850, SOVF_067770, SOVF_075310, SOVF_076310, SOVF_093600, SOVF_201960). The detection of these components suggests a potential role of PDNVs in maintaining proteostasis, consistent with reports of proteasome activity in plant vesicles during stress adaptation [22]. A substantial number of proteins involved in photosynthesis were also detected, including large and small subunits of ribulose-1,5-bisphosphate carboxylase/oxygenase (RuBisCO; rbcL, SOVF_008110, SOVF_080400, SOVF_080410, SOVF_080380), phosphoglycerate kinase (SOVF_007410, SOVF_007400), and glyceraldehyde-3-phosphate dehydrogenase (SOVF_029640, SOVF_158250, SOVF_178150). Although these enzymes are typically localized in the chloroplast, their detection in PDNVs may reflect active metabolic signaling or the transport of enzymatic machinery between subcellular compartments and/or different plant cells [23]. The category of amino acid metabolism featured enzymes involved in nitrogen assimilation and amino acid biosynthesis, such as cysteine synthase (SOVF_148110, SOVF_198480), glutamine synthetase (SOVF_156520), and glutamate dehydrogenase (SOVF_104730). This suggests a potential role for nanovesicles in nitrogen remobilization or amino acid recycling, in agreement with emerging evidence supporting the involvement of PDNVs in cellular metabolic reprogramming [24]. Proteins associated with carbohydrate metabolism included glycosyl hydrolases (SOVF_014560), phosphomannomutase (SOVF_054170), and alpha-amylase (SOVF_094290). These proteins may support sugar mobilization and energy redistribution, potentially contributing to cell wall remodeling or signaling. The glutathione metabolism group included key antioxidant proteins, such as superoxide dismutase [Cu-Zn] (SOVF_040980), glutathione reductase (SOVF_069250), and glutathione S-transferases (SOVF_100390, SOVF_151080), which could also be classified under the functional category of response to stimulus. These findings further support the idea that PDNVs play a central role in maintaining redox homeostasis and cellular protection, in accordance with previous reports on vesicles isolated from *B. vulgaris* juice, showing antioxidant and nitrite-reducing properties [25]. Additional functional categories such as protein metabolism, cell wall organization, lipid metabolism, and chromatin organization were less represented, but remain biologically relevant. For example, the presence of histone H2A (SOVF_029920) and histone H4 (SOVF_000690) suggests a potential role of PDNVs in epigenetic regulation. Similarly, detection of malic enzyme (SOVF_106750) and lipases points to a possible involvement of PDNVs in lipid remodeling and energy generation. Altogether, these findings indicate that *B. vulgaris* PDNVs are not passive carriers but active participants in plant communication. Their protein cargo reflects a coordinated strategy for defense, metabolic adaptation, and systemic signaling. The authors wish to specify that in this study, which was designed as a descriptive characterization of a single biological condition, two independent biological replicates were analyzed. Results were highly consistent (>90% of protein identity coincidence among analyses), supporting the reliability of the findings and providing a solid basis for future studies involving comparative analyses of a higher number of biological replicates.

### 2.3. Lipidomic Analysis

**Figure 5:**
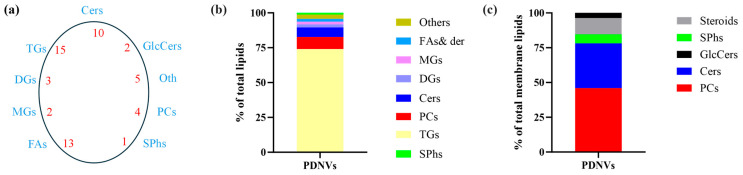
Lipid classes identified by the LC-MS/HRMS lipidomics workflow in *B. vulgaris* PDNVs. (**a**) Molecular classes were annotated according to the databases present in Lipid Search as a key node for the identification schema. The elution profile and retention times were matched with the reference analytical standards described in the experimental section. Blank samples were used for background removal. Chemical structures proposed in Lipid Search were further checked for annotation using the fragment ion search score (FISh) with a mass tolerance of 2.5 ppm, and analytical information are reported in Supplementary Table S2. (**b**) Concentration in ng/mL and ng/10^10^ particles was reported as the average of two biological replicates and the percentage toward the overall lipid identified and quantified (Supplementary Table S2). (**c**) Relative concentration of membrane lipids was reported as the average of two biological replicates and the percentage toward the overall membrane lipids identified. Abbreviations: Cers, Ceramides; GlcCers, Glucosylceramides; PCs, Phosphocholines; SPhs, Sphinganine & sphingosine; FAs, Fatty acids and derivatives, MGs, Monoacylglycerols; DGs, diacylglycerols; TGs, triacylglycerols.

The lipid composition of PDNVs is in most cases unknown, despite it is recognized that lipids absolve key structural and functional tasks. Total lipids from purified *B. vulgaris* PDNVs were extracted in acid chloroform/methanol and analyzed by liquid chromatography high-resolution tandem mass spectrometry (LC-HR-MS/MS). Fifty-five species were identified, grouped in 9 classes, and quantified according to pure reference standards and chemical class similarities (Figure 5a). These included triacyclglycerols, diacylglycerols, monoalkylglycerols, phosphocholines, ceramides, glucosylceramides, sphinganine and sphingosine, fatty acids and derivatives, and steroids and triterpenoids (Oth). The most abundant were triacyclglycerols, which represented almost 75% of total lipids. (Figure 5b). Among the membrane lipids, the class most represented was that of phosphocholines, followed by ceramides and glucosylceramides, including sphinganine and sphingosine (Figure 5c). The complete list of identified lipids is reported in Table S2.

### 2.4. Effect of B. vulgaris PDNVs on Hakata Cell Viability

**Figure 6:**
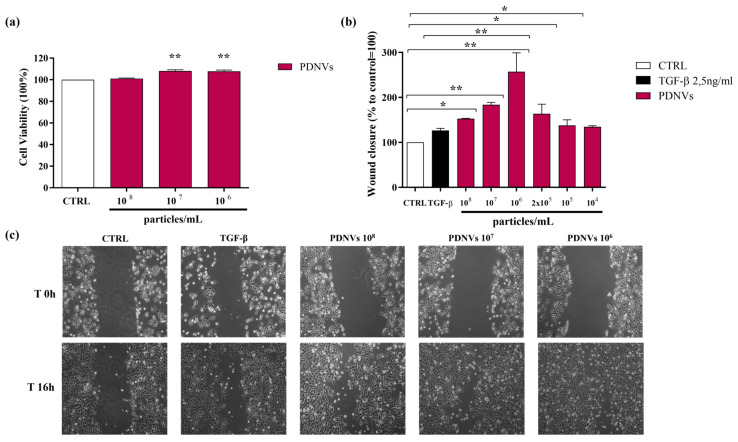
Effect of purified *B. vulgaris* PDNVs on Hakata cell viability (**a**) and wound healing (**b**,**c**). (**a**) Hakata cells were treated with PDNVs at concentrations of 10^8^, 10^7^, and 10^6^ particles mL^−1^ for 48 h and then the cell viability was measured. Data are presented as percentages relative to untreated controls. (**b**) Bar graph illustrating the percentage of wound closure at the different PDNV concentrations. Wound closure was quantified using ImageJ software (v1.38e), and the untreated group serving as the control. The bars represent the mean of three biological replicates (n = 3) ± SEM, and asterisks denote statistically significant differences determined by one-way ANOVA followed by Tukey’s test (* *p* < 0.05, ** *p* < 0.01). (**c**) Optical microscopy images of Hakata cells treated with varying PDNV concentrations, showing the scratched area. The images were taken at 0 and 16 h after treatment using a 10× optical microscope.

To evaluate the potential cytotoxicity of *B. vulgaris* PDNVs in human cells, Hakata keratinocytes were incubated for 24 h in DMEM with different concentrations of purified PDNVs (10^8^, 10^7^, and 10^6^ particles mL^−1^). Cell viability was assessed using the MTT assay. The results showed that PDNVs did not have any cytotoxic effects at the concentrations tested (Figure 6, panel a).

### 2.5. Effect of B. vulgaris PDNVs on Wound Healing in Hakata Cells

The movement of skin cells toward the wound area is a crucial phase in the wound healing process. To assess the impact of purified *B. vulgaris* PDNVs on this process, a scratch assay was performed on Hakata cells, which were subsequently treated with varying concentrations of nanovesicles (10^8^, 10^7^, 10^6^, 2 × 10^5^, 10^5^, and 10^4^ particles mL^−1^). Sixteen hours after PDNV treatment, the extent of wound closure was evaluated. *B. vulgaris* PDNVs enhanced wound closure across all tested doses (Figure 6, panel b), with the most marked effect at a concentration of 10^6^ particles mL^−1^, which was approximately twice that of TGF-β, used here as a positive control. Although the response was less pronounced at lower concentrations (10^5^ and 10^4^ particles mL^−1^), the improvement in wound repair remained statistically significant compared to the untreated control. These findings suggest that PDNV treatment effectively promoted keratinocyte movement, a fundamental mechanism involved in tissue regeneration and wound healing (Figure 6, panel c).

### 2.6. Effect of B. vulgaris PDNVs on the Transcription of Wound Healing-Related Genes in Scratched Hakata Cells

**Figure 7:**
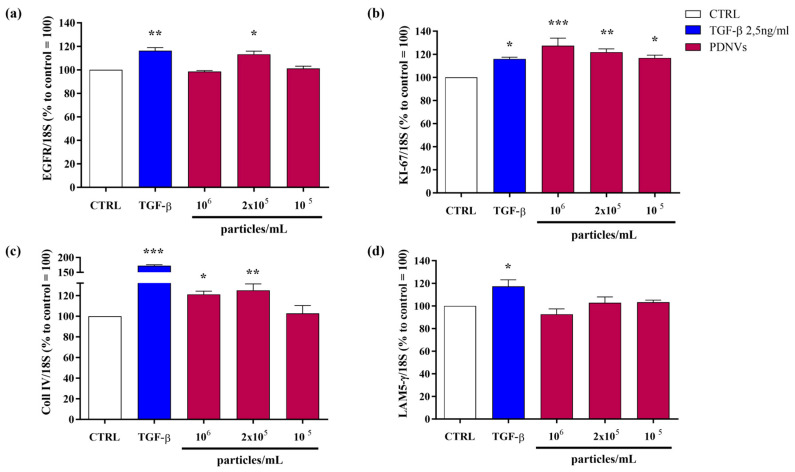
Semi-quantitative RT-PCR analysis of wound healing-related gene expression in HaCaT cells treated with *B. vulgaris* PDNVs after scratch injury. (**a**) *EGFR*, (**b**) *KI-67*, (**c**) *Coll IV*, and (**d**) *LAM5-γ* gene expression. Gene expression levels were measured by semi-quantitative RT-PCR and normalized to the 18S housekeeping gene. Results are presented as percentages relative to untreated HaCaT cells. Data represent the mean ± SEM of two independent biological replicates (n = 2). Statistical comparisons were performed using an unpaired two-tailed Student’s *t*-test (* *p* < 0.05, ** *p* < 0.01, *** *p* < 0.001).

To explore the molecular mechanisms behind the wound healing effects of purified *B. vulgaris* PDNVs, we analyzed the expression of genes involved in wound repair in scratched Hakata keratinocytes treated with PDNVs, using semi-quantitative RT-PCR. As shown in Figure 7, panel a, exposure to PDNVs at a concentration of 2 × 10^5^ particles mL^−1^ led to a significant upregulation of the *EGFR* gene, comparable to the increase induced by the positive control, TGF-β. Figure 7, panel b illustrates the effect of PDNV treatment on the transcription of the proliferation marker gene *KI-67*. The treatment with PDNV increased *KI-67* transcription, with the highest effect (130%) observed at 10^6^ particles mL^−1^ compared to the untreated group, exceeding the effect of TGF-β (118%). We also assessed the impact of PDNVs on genes associated with extracellular matrix formation, specifically collagen type IV (*Coll IV*) and laminin subunit 5 γ (*LAM5-γ*), which play roles in cell adhesion and migration. As shown in Figure 7, panel c, *Coll IV* expression was significantly increased after PDNV treatment at 10^6^ and 2 × 10^5^ particles mL^−1^, while *LAM5-γ* expression did not show significant change (Figure 7, panel d). These findings indicate that *B. vulgaris* PDNVs promote wound healing, at least in part by enhancing the expression of genes involved in cell proliferation and extracellular matrix remodeling.

### 2.7. Effect of B. vulgaris PDNVs on Nitric Oxide Production in LPS-Stimulated RAW 264.7 Cells

**Figure 8:**
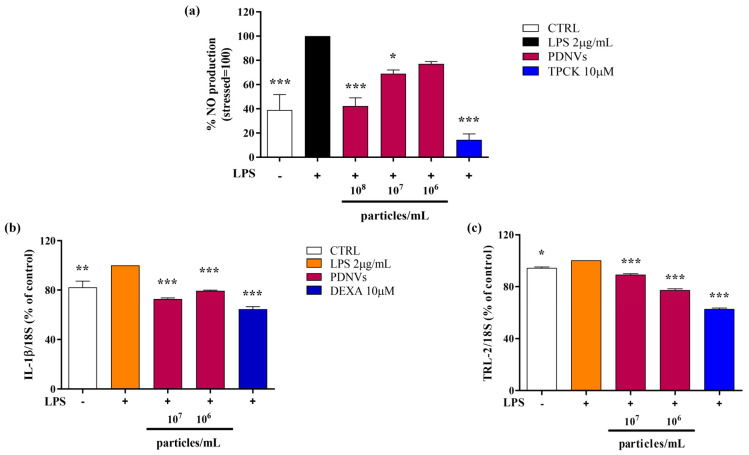
Effect of treatment with *B. vulgaris* purified PDNVs on the inhibition of NO production in LPS-stimulated RAW 264.7 cells (**a**) and on the expression of inflammation-related genes in LPS-stimulated HaCaT cells (**b**,**c**). (**a**) Bar chart of NO levels in LPS-stimulated RAW 264.7 cells, pre-treated with PDNVs for 2 h. TPCK, N-tosyl-L-phenylalanine methyl ketone; bars represent the mean of three biological replicates (n = 3) ± SEM, and asterisks denote statistically significant differences determined by one-way ANOVA followed by Tukey’s test (* *p* < 0.05, *** *p* < 0.001). (**b**,**c**) Semi-quantitative RT-PCR analysis of the pro-inflammatory genes *IL-1β* (**b**) and *TLR2* (**c**) in LPS-stimulated HaCaT cells. Data represent the mean ± SEM of two independent biological replicates (n = 2). Statistical comparisons were performed using an unpaired two-tailed Student’s *t*-test (* *p* < 0.05, ** *p* < 0.01, *** *p* < 0.001). Dexa: dexamethasone.

The bacterial lipopolysaccharide (LPS) is a pro-inflammatory stimulus able to strongly induce nitric oxide (NO) production, which subsequently promotes the excessive generation of inflammatory cytokines and reactive oxygen species (ROS). To assess the impact of purified *B. vulgaris* PDNVs on NO production under inflammatory conditions, RAW 264.7 cells were pre-treated with PDNVs at concentrations of 10^8^, 10^7^, and 10^6^ particles mL^−1^ and then stimulated with 2 μg mL^−1^ LPS. Nitrite levels in the culture medium, which served as an indicator of NO production, were measured spectrophotometrically at 340 nm using the Griess reagent. As illustrated in Figure 8, panel a, pre-treatment with PDNVs significantly decreased NO production compared to LPS-stimulated cells without PDNV pre-treatment. Interestingly, a concentration of 10^8^ particles mL^−1^ reduced the NO levels by 50%, an effect of the same extent as that produced by TPCK, a serine protease inhibitor known to reduce inflammation by blocking NF-κB activation.

### 2.8. Effect of B. vulgaris PDNVs on the Transcription of Inflammation-Related Genes in LPS-Stimulated HaCaT Cells

To evaluate the capability of purified *B. vulgaris* PDNVs to downregulate the transcription of pro-inflammatory genes, such as the interleukin-1β gene (*IL-1β*), and the Toll-like receptor 2 gene (*TLR-2*), HaCaT cells were pre-incubated with 10^7^, and 10^6^ particles mL^−1^ of purified PDNVs and then treated with 2 μg mL^−1^ LPS. Results of semi-quantitative RT-PCR showed that PDNV pre-treatment reduced *IL-1β* transcription compared to the non-treated cells (Figure 8, panel b). The effect was similar at both concentrations and lower than that induced by the anti-inflammatory positive control, dexamethasone (DEXA) at 10 μM. On the contrary, the downregulation of *TLR-2* transcription was more markedly affected and in a dose-dependent way (Figure 8, panel c).

### 2.9. MicroRNA Analysis and Bioinformatic Prediction of Potential Human Gene Targets

Growing evidence suggests that PDNVs can modulate mammalian gene expression [9]. To investigate this possibility, we examined the miRNA profile of purified *B. vulgaris* PDNVs, employing a high-throughput sequencing approach. The expression profile was generated by mapping the sequences to the miRBase reference database (v22.1). In total, 30 miRNAs were identified, including 17 unique sequences. These miRNAs, along with their corresponding read counts, are reported in Table 1.

To explore the potential ability of identified miRNAs to modulate human gene expression, we performed a bioinformatic analysis using the psRNATarget algorithm to predict their putative targets. For each miRNA, the most likely target genes, determined by expectation scores, are listed in Table 2, while the complete set of predicted human targets is provided in Table S3. In total, 23 human genes were identified as the most likely targets of *B. vulgaris*-derived miRNAs.

**Figure 9:**
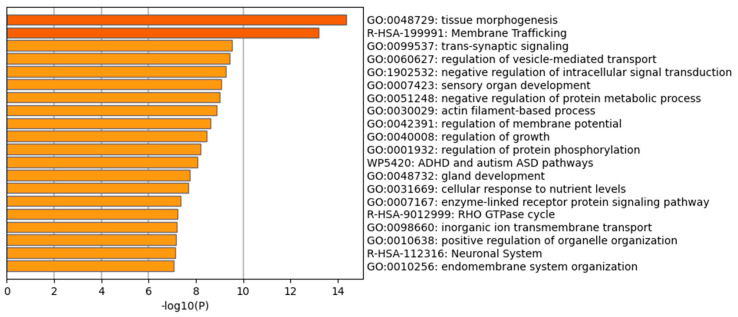
GO/KEGG functional enrichment analysis performed using the predicted human target genes of miRNAs identified in *B. vulgaris* PDNVs. The bar chart displays Gene Ontology (GO) terms as well as Wiki and Reactome pathways derived from the enrichment analysis. Biological processes and pathways are ranked by *p*-value, from the most significant (dark orange) to the least significant (light orange).

Overall, the results in Table 2 suggest the potential of *B. vulgaris* microRNAs to regulate human genes associated with biological processes related to tissue repair, such as cytoskeletal organization and remodeling, actin organization, cell division, proliferation and mobility, as well as processes linked to the inflammatory response, including vesicular trafficking and the regulation of inflammatory and immune responses. To further explore the functional implications of the predicted targets, an enrichment analysis was performed using Gene Ontology (GO) and KEGG annotations of cellular processes. The GO and KEGG terms were selected based on cumulative hypergeometric *p*-values and enrichment factors. Hierarchical clustering was conducted with a kappa score threshold of 0.3. Figure 9 shows a bar chart summarizing the GO pathway enrichment analysis. Tissue morphogenesis, a key process critical for cellular organization and tissue regeneration, exhibited the highest enrichment score. Other significantly enriched pathways relevant to tissue regeneration included membrane trafficking and actin filament-based process. Furthermore, regulation of vesicle-mediated transport suggests that these miRNAs may modulate the dynamics of secretory and endocytic vesicles, which are essential for cargo delivery, signal transmission, and coordination of cell–cell interactions. GO and KEGG pathway enrichment analyses confirmed that *B. vulgaris*-derived miRNAs have the potential to regulate pathways associated with cell differentiation and tissue organization, highlighting their possible involvement in PDNV-mediated stimulation of tissue repair.

## 3. Discussion

Improving the characterization of PDNV structural components and cargo molecules is a crucial step toward more targeted and effective applications. In this study, taking advantage of an innovative procedure previously used to isolate PDNVs from *B. oleracea* [18], we successfully purified PDNVs from *B. vulgaris*, a widely cultivated plant species whose biological properties remain largely underexplored. Results from the NTA analysis indicated that our platform produced concentrated (about 2 × 10^10^ particles mL^−1^) and homogeneous PDNV preparations of purity comparable to that of mammalian nanovesicles used in therapeutic applications [16,26]. TEM imaging showed that purified PDNVs remained structurally intact, an essential condition for biological activity. The availability of sufficient amounts of highly purified *B. vulgaris* PDNVs enabled their extensive -omic characterization.

STRING analysis of the cargo proteins identified by mass spectrometry revealed their functional enrichment in 14 different categories of biological processes. The protein profile was consistent with those reported in previous studies for PDNVs from various sources [21,27,28,29,30,31,32,33], confirming the selective enrichment of conserved protein classes that reflect the functional roles of PDNVs in recipient plant cells. Overall, the most represented proteins were those involved in response to stimulus, particularly oxidative stress; the concomitant occurrence of the functionally related classes of glutathione metabolism, proteasome, and cell wall organization strongly suggests that a crucial role of PDNVs in plants is the regulation of redox and protein homeostasis during adaptation to abiotic or biotic stress. The identification of a variety of proteins capable of regulating redox homeostasis may partly explain the anti-inflammatory activity reported for PDNVs in human cells [11], and their characterization provides a valuable foundation for future studies aimed at elucidating the underlying molecular mechanism(s).

Research on the lipid composition of PDNVs remains limited, although lipids are key structural components of membranes and can affect nanovesicle stability, transport, uptake, and function. Hence, the determination of the PDNV lipid profile can help to shed light on the biochemical pathways underlying bioactivities. Results from lipidomic analysis showed that the most abundant membrane lipids in *B. vulgaris* PDNVs were phosphocolines followed by sphingolipids, including ceramides, glucosylceramides, sphinganine, and sphingosine. From the up-to-date available literature data, phospholipids, including phosphocolines, are the most abundant species in various plants [34] and have been shown to modulate PDNV targeting and tissue repair activity [35,36]. Sphingolipids have been detected as abundant components of PDNVs isolated from *A. thaliana* [37] and *B. oleracea* plants [18], but data regarding their involvement in the modulation of PDNV targeting and/or functions are lacking, although in animal cells, sphingolipids are involved in the modulation of key processes including proliferation and inflammation [38].

To investigate the therapeutic potential of *B. vulgaris* PDNVs, we evaluated their wound healing and anti-inflammatory activities in vitro using human cells. The results showed that PDNVs effectively promoted wound closure in scratched HaCaT cells. This effect was correlated with the up-regulation of genes associated with key wound healing processes, as revealed by semi-quantitative RT-PCR. In particular, increased expression was observed for *EGFR* and *KI-67*, both genes involved in cell proliferation, and *Coll IV*, involved in cell migration and adhesion. The ability of PDNVs to stimulate cell proliferation has been previously reported for nanovesicles isolated from various plant sources [18,27,39,40,41]. However, the molecular pathways underlying this effect remain largely undetermined, with the exception of wheat-derived PDNVs, where epithelial stem cell proliferation was linked to activation of the Wnt/β-catenin signaling pathway [27]. The stimulatory effect of PDNVs on cell migration has also been described for nanovesicles isolated from other plant sources [26,42], and has been associated with the increased secretion of collagen I, a component of the cellular matrix necessary for cell migration [43].

The anti-inflammatory activity of PDNVs was assessed by measuring their ability to reduce NO production in LPS-stimulated human cells. The results showed that PDNVs significantly decreased the LPS-induced NO levels. This finding was supported by semi-quantitative RT-PCR analysis, which revealed the dow-nregulation of the pro-inflammatory cytokine gene *IL-1β* and the Toll-like receptor-2 gene (*TLR-2*). These results are consistent with our previous findings using PDNVs from *B. oleracea* [18] as well as those of You and coworkers, who showed that cabbage-derived PDNVs downregulated the expression of pro-inflammatory *IL-1β*, *IL-6* and *COX-2* genes in LPS-stimulated cells [39].

Growing evidence suggests that miRNAs carried by PDNVs are delivered into mammal tissues, where they regulate the expression of genes involved in different cellular processes [11,12,32,34], including tissue regeneration [44] and inflammation [45]. Therefore, we characterized the cargo miRNA of purified nanovesicles and conducted a bioinformatic analysis to predict the human genes potentially targeted by these miRNAs. The analysis identified several candidate target genes involved in biological processes related to tissue repair and the regulation of inflammation. Specifically, five genes were linked to key aspects of tissue repair: three genes associated with cytoskeletal organization and remodeling (*PPM1E, C12orf74, SYNPO2*), and two genes involved in cell proliferation and motility (*ARL4C, MKL2*). Additionally, one gene (*SERPINB8*) was associated with anti-inflammatory response, and another one (*C10orf10*) with autophagy in response to oxidative stress. Overall, *B. vulgaris* PDNVs were found to contain various miRNAs that may regulate human genes involved in wound healing and inflammatory pathways, providing useful insights to guide future research.

In conclusion, in this study, employing an innovative anion exchange-based method for isolating PDNVs, a thorough molecular characterization of *B. vulgaris* nanovesicles using proteomics, lipidomics, and miRNomics was carried out. Multiple classes of bioactive cargo molecules, including proteins, lipids, and miRNAs, were identified, which may explain the in vitro tissue repair and anti-inflammatory effects of PDNVs. These results offer a solid foundation for future investigations aimed at understanding the mechanisms through which PDNVs act in human cells.

## 4. Materials and Methods

### 4.1. Chemicals

All chemicals used for proteomic and LC-HR-MS/MS analysis, including acetonitrile, water, methanol, chloroform, isopropanol, and formic acid were of mass spectrometry-grade and sourced from Merck (Darmstadt, Germany). Analytical standards such as phosphocholine, triglycerides, diglycerides, and ceramides were purchased from Avanti (Birmingham, AL, USA), while phytochemicals, asiatic acid, and stigmasterol were sourced from Merck (Darmstadt, Germany). Reagents for SDS-PAGE were obtained from Bio-Rad (Hercules, CA, USA). All other chemicals were of analytical grade and also purchased from Merck (Darmstadt, Germany).

### 4.2. Plant Materials and Growth Conditions

Seeds of *Beta vulgaris* L., var. *Italica* were purchased from Natures Root (www.naturesroot.co.uk). These were grown in a controlled climate chamber (VB1514 Vötsch, Rosenfeld, Germany) maintained at 22 °C with 80% relative humidity and a 16/8 h light/dark cycle, as previously reported [18]. After two weeks, the above-ground portions of the seedlings were harvested and collected for PDNV isolation.

### 4.3. Preparation of the PDNV Extract for Anion Exchange Chromatography

One hundred grams of the aerial portions of *B. vulgaris* seedlings were collected and homogenized in a Waring blender, in 100 mL of phosphate-buffered saline (PBS, pH 7.4) containing 1 mL of Protease Inhibitor Cocktail (Thermo Fisher Scientific, Waltham, MA, USA), 1 mM sodium azide, 1 mM phenylmethylsulphonyl fluoride, and 1 mM leupeptin. The resulting homogenate was then filtered through cheesecloth and centrifuged at 8000× *g*, and 18,000× *g* for 30 min. The resulting supernatant was sequentially filtered, concentrated by tangential flow filtration, and treated with micrococcal nuclease (MNase, Thermo Fisher Scientific, Waltham, MA, USA), as previously reported [18]. After nuclease digestion, the sample was diafiltered against 50 mM HEPES, 180.7 mM NaCl, pH 7.4 [17], and subjected to anion exchange-FPLC purification, as previously reported [18]. All procedures were carried out with samples and buffers maintained at 4 °C.

### 4.4. Anion Exchange Chromatography Purification of PDNVs

Anion exchange chromatography was carried out on an AKTA FPLC system (Cytiva, Marlborough, MA, USA) with a CIMmultus™ EV monolith column (1 mL, Sartorius, Göttingen, Germany) according to the conditions described by Zanotti and coworkers [18]. Fractions of 2 mL were analyzed for protein content using the Bradford assay [46] and for PDNV detection by NTA. Fractions containing PDNVs were pooled and used for analysis or stored at −20 °C until use.

### 4.5. Nanoparticle Tracking Analysis

The size distribution and concentration of purified PDNVs were determined using nanoparticle tracking analysis (NTA) on a NanoSight NS300 instrument (Malvern Instruments, Malvern, UK). PDNV samples were diluted 100- to 500-fold in PBS to achieve a particle concentration in the range of 10^8^–10^9^ particles mL^−1^. Measurements were performed by scanning 11 different positions in the sample cell in scatter mode, capturing 60 frames per position using a 532 nm laser and a sCMOS camera (Thorlabs, Newton, NJ, USA). The camera level was set at 14 for maximum sensitivity with a minimum of background noise, and the detection threshold was set to 5. For each sample, three videos of 30 s each were recorded and analyzed using Nanoparticle Tracking Analysis software version 3.4 (Malvern Instruments, Malvern, UK). The area under the histogram was evaluated in triplicate for each sample, and the average value was used to represent the particle concentration. Three independent experiments were conducted. All NTA analyses were performed using identical instrument settings to maintain consistency. The experiments were carried out at the Core Facilities of the Istituto Superiore di Sanità, Rome, Italy.

### 4.6. Transmission Electron Microscopy

PDNV samples were subjected to negative staining following the procedure described previously [18]. Briefly, PDNVs (with concentrations >10^10^ before and >10^7^ after sorting, as measured by NTA) were resuspended in 100 µL of PBS, and 10 µL of this suspension was placed on formvar-carbon coated grids. To enhance contrast, 5 µL of 4% (*w*/*v*) ammonium molybdate at pH 6.4 was applied for 30 s and then removed using filter paper. The grids were allowed to air dry and subsequently visualized using a PHILIPS EM208S transmission electron microscope (FEI, Thermo Fisher Scientific, Waltham, MA, USA) at the Department of Physics, Istituto Superiore di Sanità, Rome, Italy.

### 4.7. SDS-PAGE and Western Blotting

SDS-PAGE analysis was performed using a Mini Protean II Dual SLAB Cell system (Bio-Rad, Hercules, CA, USA) according to Laemmli [47]. Proteins were visualized by staining with 0.1% (*w*/*v*) Coomassie Blue (Bio-Rad). After electrophoretic separation, proteins were transferred onto a polyvinylidene fluoride (PVDF) membrane (Bio-Rad, Hercules, CA, USA ) using a Trans-Blot semi-dry transfer apparatus (Bio-Rad), as previously described [48]. The membrane was incubated with anti-TET8 primary antibody (PhytoAB, San Josè, USA, code PHY3337A) and then with horseradish peroxidase conjugated secondary antibody (anti-mouse, 1:10,000; Bio-Rad, Hercules, CA, USA), as previously described [18]. Antigen–antibody binding was detected by luminol chemiluminescence (Euroclone, Milan, Italy), as reported in [18], and chemiluminescent signals were captured using the VersaDoc™ 4000 MP imaging system (Bio-Rad, Hercules, CA, USA).

### 4.8. Protein Extraction

Purified PDNVs from two independent biological samples of *B. vulgaris* were dried in parallel on a SpeedVac rotary evaporator (Thermo Fisher Scientific, Waltham, MA, USA) and resuspended in 50 μL of lysis buffer (7 M urea, 2 M thiourea, 4% (*w*/*v*) CHAPS, and 1% (*w*/*v*) DTT) containing a plant-specific protease inhibitor cocktail (Sigma-Aldrich, St. Louis, MO, USA). The samples were subjected in parallel to ultrasonication in a water bath for three cycles of 4 min each, with 2 min cooling intervals on ice, followed by an incubation of 1 h on ice. After lysis, the samples were centrifuged at 16,000× *g* for 10 min at 4 °C, and the supernatants were collected for further analysis. Protein concentrations were determined using the Quick Start™ Bradford Protein Assay (Bio-Rad).

For preparative SDS-PAGE, 50 μg of proteins from each PDNV preparation were loaded onto 12% T gels under reducing conditions and run in parallel. Following electrophoresis, the gels were sliced into 14 equal segments, which were minced and sequentially washed with water and acetonitrile. Proteins within the gel pieces were reduced and alkylated using 55 mM iodoacetamide in 100 mM ammonium bicarbonate, followed by additional washing. Proteolytic digestion was performed overnight with trypsin (12.5 ng μL^−1^ in 50 mM ammonium bicarbonate containing 5 mM CaCl_2_). The resulting peptides were desalted and concentrated using ZipTip C18 tips (Merck), dried under vacuum, and finally resuspended in 0.1% (*v*/*v*) formic acid in preparation for further mass spectrometry analysis.

### 4.9. Proteomics

Peptide samples were analyzed using a nanoLC-ESI-Q-Orbitrap-MS/MS setup, which combined a Vanquish Neo nanoliquid chromatography system with an Exploris 480 mass spectrometer equipped with an EASY-Spray ion source (Thermo Fisher Scientific). Peptides were loaded onto an EASY-Spray C18 column (150 mm × 75 μm ID, 2 μm particle size, 100 Å pore size) and separated using a gradient elution of solvent B (80% acetonitrile, 0.08% formic acid in water) in solvent A (0.08% formic acid in water) at a flow rate of 250 nL/min. Solvent B was increased from 6% to 31% over 30 min, then 50% over 5 min, followed by an increase to 95% over 5 min, held at 95% for 4 min, and finally returned to 6% for column re-equilibration. Mass spectrometry data were acquired in data-dependent acquisition mode, with full scans performed over an *m/z* range of 375–1200 at a resolution of 120,000 (at 200 *m/z*). MS/MS spectra were generated from the 20 most intense precursor ions using a normalized collision energy (HCD) of 30%, a normalized AGC target of 200%, a maximum injection time of 100 ms, and a resolution of 30,000 (at 200 *m/z*). Dynamic exclusion was set to 45 s.

For protein identification, all raw MS data from the gel-slice digests of the same PDNV preparation were combined and processed using Proteome Discoverer v. 2.4 (Thermo Fisher Scientific, Waltham, MA, USA), with database searches performed through the Mascot algorithm v. 2.4.2 (Matrix Science, Boston, MA, USA) following a shotgun proteomics approach. The *B. vulgaris* protein database from NCBI (67,659 entries, April 2024) was used. Search settings included the carbamidomethylation of cysteine as a fixed modification and the oxidation of methionine, N-terminal pyroglutamate formation, and phosphorylation at serine, threonine, and tyrosine as variable modifications. Mass tolerances were set to ±10 ppm for precursor ions and ±0.05 Da for fragment ions. Trypsin was specified as the protease, allowing up to two missed cleavages. Proteins were considered confidently identified only if they met stringent criteria: false discovery rate (FDR) below 1%, Mascot peptide scores ≥ 25, unambiguous peptide spectrum matches (PSMs) with rank 1, and a Delta CN (difference between the top-scoring and next-best PSM) of 0. A comparison of the proteins identified in the two independent PDNV preparations demonstrated a coincidence of results > 90%, which was fully compatible with the variability of SDS-PAGE-based proteomic data using single peptide-based high-resolution mass spectrometry identifications [49,50].

### 4.10. Lipid Extraction, LC-HR-MS/MS Analysis and Untargeted Lipidomics

The Folch method was used for lipid extraction from pooled PDNV fractions purified from two independent *B. vulgaris* biological samples: (0.1 mL each) were mixed with chloroform:methanol (2/1, *v*/*v*, 2 mL) and 0.05 mL of HCl, 0.1 N. Suspensions were gently mixed, then centrifuged at 18,000× *g*, for 20 min at 4 °C according to the procedure described by Zanotti and coworkers [18]. The dried supernatant was dissolved in 80% isopropanol for LC-HR-MS/MS analysis. A Vanquish liquid chromatographic system was connected to an Exploris 120 quadrupole Orbitrap high-resolution mass spectrometer (Thermo Fisher Scientific, Waltham, MA, USA). Liquid chromatography separation encompassed the use of a C18 core shell column (Kinetex C18 PS, 100 × 2.1, 2.6 µm, Phenomenex, Torrance, CA, USA), which was eluted at 0.2 mL/min flow with the following gradient of solvent B (5 mM ammonium formate with 0.1% formic acid in isopropanol: acetonitrile 90/10 *v*/*v*) in solvent A (5 mM ammonium formate with 0.1% formic acid in water:acetonitrile 60/40 *v*/*v*): (minutes/%B), (0/20), (0.5/20), (3/50), (10/70), (18/97), (22/97) at 50 °C. Full-scan acquisition runs in polarity switching mode scanned the ions in the *m/z* range 120–1500. Heated electrospray interface (H-ESI) static spray voltage was −3.2 kV in negative ions and 3.5 kV for positive ions; ion transfer tube temperature, 300 °C; vaporizer temperature, 320 °C; sheath gas flow, 50 arbitrary units; auxiliary gas flow, 10 arbitrary units. The analyzer resolution was set at 60,000 (FWHM at *m/z* 200) and data were acquired with a normalized all-gain control (AGC) target of 100%. Data-dependent scanning experiments (ddMS^2^) covered differential scan ranges by working alternatively in positive or in negative ion mode (*m/z* 120–450, *m/z* 440–750, *m/z* 740–1050, *m/z* 1040–1500, resolution 60000, FWHM at *m/z* 200). Profile data were analyzed using Xcalibur 4.5 (Thermo Fisher Scientific, Waltham, MA, USA), fragmentation spectra were recorded using Free Style software (v. 1.8, Thermo Fisher Scientific, Waltham, MA, USA), and analytical performances matched those reported by Zanotti and coworkers [18]. An untargeted lipidomic workflow based on the identification of phytochemicals, primary, and secondary apolar metabolites was used by using differential scanning ranges as identification quality control runs in Compound Discoverer (v. 3.3, Thermo Fisher Scientific, Waltham, MA, USA). Along with the *m/z* exclusion list based on procedural blank samples, the multivariate filtering procedure was applied to background removal, presence of normalized area, presence of the MS^2^ dimension, and presence of the molecular formula. Identification was accomplished through the isotopic pattern distribution and fragmentation spectra against the MS fragmentation pattern available in databases as mzCloud, ChemSpider, natural compounds Atlas, LipidMaps, and KEGG. Specifically, an internal mass list was generated starting with the LipidSearch structure database that was used as a primary reference for the compound annotation; each screened molecule was then fragmentated in silico by using the FISh (fragment ion search) score combined with sequential elution profiles. Wherever possible, identification of annotated compounds was further confirmed through the analysis of available standards present in the LightSPLASH^TM^ quantitative mass spectrometry primary standards (Avanti, Birmingham, AL, USA) and by using diagnostic ions reported in both publicly available databases and in the study of Claassen and coworkers [51]. Calibration curves were performed by using reference standards in LightSPLASH^TM^ reported in bold in Supplementary Table S2 along with asiatic acid and stigmasterol. Analytical performances were monitored through the interday and intraday assay by comparing the slopes of each calibration curve replicated within TraceFinder quantitation software (v. 5.2, Thermo Fisher Scientific, Waltham, MA, USA). Final concentration was reported as ng/mL and as ng/10^10^ particles.

### 4.11. Total RNA Extraction

Anion exchange-purified *B. vulgaris* PDNVs (400 µL) were mixed with 1 mL of NucleoZOL (MACHEREY-NAGEL, Düren, Germany) and 400 µL RNase-free water, vortexed, and centrifuged at 12,000× *g* for 10 min, at room temperature. The aqueous phase was collected, and RNA was precipitated with an equal volume of isopropanol, washed twice with 75% ethanol, and resuspended in 25 µL RNase-free water. RNA concentration was measured using a NanoDrop spectrophotometer (Thermo Fisher Scientific, Waltham, MA, USA). Three biological replicates were processed for subsequent miRNA sequencing.

### 4.12. Small RNA Sequencing

RNA quality was evaluated using a Qubit 4.0 fluorometer (Invitrogen, Waltham, MA, USA) and TapeStation 4200 (Agilent, Santa Clara, CA, USA). Indexed libraries were generated from 5 µL RNA, using the QIAseq^®^ miRNA UDI Library Kit (Qiagen, Venio, The Netherlands) following the manufacturer’s protocol. Libraries were quantified with Qubit and TapeStation, pooled in equimolar amounts, and sequenced on an Illumina NovaSeq 6000 (Illumina, San Diego, CA, USA), using a 1 × 75 single-end run.

### 4.13. Bioinformatic Analysis for microRNA Identification

Raw small RNA sequencing data were assessed for quality using FastQC (v0.12.1) [52] to detect potential issues with the sequencer or library. Reads were evaluated for quality and adapter contamination, and low-quality reads and adapters were removed using sRNAbench [53] with the Qiagen QIAseq miRNA protocol. MicroRNA expression profiles were generated by mapping reads to reference sequences from miRBase v22.1 [54], using *A. thaliana* as a surrogate genome for *B. vulgaris*. Expression levels were normalized to counts per million (CPM): CPM = (miRNA reads/total mapped reads) × 10^6^. Human mRNA targets of the identified miRNAs were predicted with psRNATarget [55] using the *H. sapiens* transcriptome. Functional enrichment of predicted targets, including biological processes, molecular functions, and cellular components, was analyzed via Gene Ontology using Metascape [56].

### 4.14. Skin Cell Cultures

Immortalized Human Keratinocytes (HaCaT), purchased from Addexbio Technologies (San Diego, CA, USA), were maintained under conditions previously described [18]. Murine RAW 264.7 macrophages were obtained from the European Collection of Cell Cultures (ECACC; Health Protection Agency Culture Collection, Porton Down, UK). Cells were cultured in DMEM (Gibco, Frederick, MD, USA) and maintained under conditions previously described [18]. All in vitro experiments were conducted at Arterra Bioscience, Naples, Italy.

### 4.15. Treatment of Skin Cell Lines with PDNVs

HaCaT and RAW 264.7 cells, cultured in DMEM with 10% FBS and 1% penicillin-streptomycin at 37 °C, 5% CO_2_ were used for experiments at 70–80% confluence. Cells were treated with fresh media containing 1 × 10^7^ or 1 × 10^6^ particles mL^−1^ of purified PDNVs, or with TGF-β or dexamethasone as positive controls.

### 4.16. Cytotoxicity Assay

Cytotoxicity was assessed using the MTT assay. Cells were seeded in 96-well plates in DMEM with 10% FBS and incubated for 8 h. These were then treated with purified PDNVs (1 × 10^8^, 1 × 10^7^, or 1 × 10^6^ particles mL^−1^) for 48 h. After washing with PBS, cells received 100 µL/well MTT buffer (0.5 mg/mL MTT in PBS with HEPES, CaCl_2_, MgSO_4_, and glucose) and were incubated for 3 h at 37 °C, 5% CO_2_. Next, 100 µL solubilizing solution (10% Triton X-100, 0.1 N HCl in isopropanol) was added and incubated for 16 h. Absorbance was read at 595 nm using a Victor3 Nivo plate reader (PerkinElmer, Waltham, MA, USA).

### 4.17. Scratch Assay

HaCaT cells were seeded in 6-well plates (8 × 10^5^ cells/well). After 6 h, a scratch was made in each well forming similar cell-free zones, and detached cells were removed with PBS. Cells were then incubated for 7 h in 0.5% FBS medium with or without PDNVs (1 × 10^8^, 1 × 10^7^, or 1 × 10^6^ particles mL^−1^); TGF-β (2.5 ng/mL) served as a positive control. Scratch area was quantified at 0 and 16 h using ImageJ. The decrease in scratch area was interpreted as an integrated wound-healing response, reflecting the combined effects of migration and/or proliferation.

### 4.18. Analysis of Gene Expression in Scratch-Wounded HaCaT Cells

Total RNA was extracted from scratch-wounded HaCaT cells treated with *B. vulgaris* PDNVs or left untreated, as reported by Zanotti and coworkers [18]. Briefly, RNA was purified using the GenElute™ Kit and treated with DNase I (37 °C, 30 min) to remove genomic DNA. Then, 500 ng of RNA was reverse-transcribed using Reverse Transcriptase. Semi-quantitative RT-PCR was performed with universal 18S primers as internal controls. PCR products were resolved on 1.5% agarose gels and imaged using the iBright system. Each group was processed in triplicate, with two independent sets used for semi-quantitative RT-PCR. Primer sequences were as follows:
**Primers****Sequence**EGFR FwATGTCCTCATTGCCCTCAEGFR RvCACATCCATCTGGTACGTKi-67 FwCTAGAAATCGAACACCAGCKi-67 RvGGATTTCTGAACCTGACTCColl IV FwTGTGACCAGGCATAGTCAColl IV RvGAGCCAAAAGCTGTAAGCLAM5-γ FwATCAACAGGTGAGCTATGGLAM5-γ RvCAATCTCCTGTGTCTGGAT

### 4.19. Nitric Oxide Assay in LPS-Stimulated RAW 264.7 Murine Macrophages

Nitric oxide production in RAW 264.7 cells (1.5 × 10^5^ cells/well, 96-well plates) was measured after 24 h of incubation. Experiments were performed as described by Zanotti and coworkers [18]. Briefly, cells were pre-treated for 2 h with varying concentrations of purified *B. vulgaris* PDNVs or 10 μM TPCK (positive control), then stimulated with LPS (2 μg/mL) for 18 h. Nitrite levels were quantified using the Griess reagent, and absorbance was read at 540 nm on a Victor3 Nivo plate reader (PerkinElmer).

### 4.20. Analysis of Inflammatory Genes Expression in LPS-Stimulated HaCaT Cells

HaCaT cells (1.5 × 10^5^/well) were seeded in 6-well plates for 8 h, then treated overnight with purified PDNVs or 10 μM dexamethasone (positive control). Inflammation was induced with LPS (5 μg/mL), and RNA was extracted 6 h later. Experiments were conducted in triplicate with two biological replicates. RNA was purified (GenElute™ Kit, Sigma-Aldrich), treated with DNase I (Thermo Scientific) for 30 min at 37 °C, and 500 ng was reverse-transcribed. Semi-quantitative RT-PCR used universal 18S primers as controls; PCR products were run on 1.5% agarose gels and imaged with the iBright system (Invitrogen). Primer sequences were as follows:
**Primers****Sequence***IL-1β Fw*ATGGCAGAAGTACCTGAGCT*IL-1β Rv*AAGGACATGGAGAACACCAC*TLR2 Fw*CTGGGCAGTCTTGAACAT*TLR2 Rv*CATCTTTTCTGGCCACTGACA

### 4.21. Statistical Analysis

Statistical analyses were performed using GraphPad Prism 9.5. Data were expressed as the mean ± SEM. The number of independent biological replicates (n) is indicated in each figure legend. Group differences were assessed by one-way ANOVA with Tukey’s post hoc test for experiments with n = 3, while comparisons between two groups (n = 2) were analyzed using an unpaired two-tailed Student’s *t*-test. A *p* value < 0.05 was considered statistically significant (* *p* < 0.05, ** *p* < 0.01, *** *p* < 0.001).

## Figures and Tables

**Table 1:** List of miRNAs purified and sequenced from *B. vulgaris* PDNVs. The miRBase database (v22.1) was used for analysis, selecting *A. thaliana* reference sequences.

miRNA Name	miRNA 5′-3′ Sequence	High Quality Reads
ath-miR159a	UUUGGAUUGAAGGGAGCUCUA	130,619
ath-miR159b-3p	UUUGGAUUGAAGGGAGCUCUU	127,618
ath-miR159c	UUUGGAUUGAAGGGAGCUCCU	104,742
ath-miR157d	UGACAGAAGAUAGAGAGCAC	87,007
ath-miR157b-5p	UUGACAGAAGAUAGAGAGCAC	67,930
ath-miR319a	UUGGACUGAAGGGAGCUCCCU	48,439
ath-miR156d-5p	UGACAGAAGAGAGUGAGCAC	37,434
ath-miR156g	CGACAGAAGAGAGUGAGCAC	25,175
ath-miR8175	GAUCCCCGGCAACGGCGCCA	8703
ath-miR156i	UGACAGAAGAGAGAGAGCAG	5847
ath-miR319c	UUGGACUGAAGGGAGCUCCUU	4335
ath-miR168a-5p	UCGCUUGGUGCAGGUCGGGAA	4198
ath-miR167d	UGAAGCUGCCAGCAUGAUCUGG	3749
ath-miR164b-5p	UGGAGAAGCAGGGCACGUGCA	3742
ath-miR164c-5p	UGGAGAAGCAGGGCACGUGCG	3385
ath-miR167a-5p	UGAAGCUGCCAGCAUGAUCUA	2873
ath-miR166a-3p	UCGGACCAGGCUUCAUUCCCC	2515

**Table 2:** List of putative human target genes predicted for the miRNAs identified in *B. vulgaris* PDNVs. For each miRNA, the most likely target genes, based on expectation values, are reported using the NCBI reference sequence and the official gene symbol. The corresponding expectation values, descriptions of biological functions, and aligned gene sequences are also included.

miRNA	Target Gene	Expectation Value	Biological Function	Gene Aligned Sequence
ath-miR159a	NM_014906|*PPM1E*	2.5	Protein Phosphatase, Mg^2+^/Mn^2+^ Dependent 1E, inhibitor of kinase activity; affects cytoskeletal remodeling and metabolic regulation.	AUGCUCUUUGUCUUUUGUCAA
ath-miR159a	NM_001024455|*RGAG4*	2.5	Retrotransposon Gag Domain Containing Protein 4; involved in the regulation of gene expression and the maintenance of genomic stability.	AUGUUUUCUGUCUUCUGUUAA
ath-miR157d	NM_001031848|*SERPINB8*	1.5	Serpin B8: member of the serine protease inhibitor (serpin) family; modulates inflammation and immune responses.	AUGCUCUUUGUCUUUUGUCA
ath-miR157d	NM_007021|*C10orf10*	2	DEPP1 (Decidual Protein Induced by Progesterone); regulates FOXO3-induced autophagy via increased cellular reactive oxygen species (ROS).	AUGUUUUCUGUCUUCUGUUA
ath-miR157d	NM_000599|*IGFBP5*	2.5	Insulin-Like Growth Factor Binding Protein 5; regulates growth, bone metabolism, and fibrotic diseases.	UUGAUCUUUGUCUUUUGUCA
ath-miR319a	NM_014906|*PPM1E*	2.0	Protein Phosphatase, Mg^2+^/Mn^2+^ Dependent 1E, inhibitor of kinase activity; affects cytoskeletal remodeling and metabolic regulation.	AAGUAGUUCCCUUCAGUUCAA
ath-miR156d-5p	NM_001178097|*C12orf74*	2.5	Chromosome 12 open reading frame 74, putative guanyl-nucleotide exchange factor (GEF) for Rho GTPases; involved in actin cytoskeleton organization, cell migration, and cell cycle progression.	UCGCUCACUUUCUUCUGUCC
ath-miR156d-5p	NM_152795|*HIF3A*	2.5	Hypoxia-Inducible Factor 3 Alpha Subunit; regulates the adaptive response to hypoxia and suppresses hypoxia-inducible gene expression.	CUGCUCGCUCUGUUUUGUCA
ath-miR156d-5p	NM_152387|*KCTD18*	2.5	Potassium Channel Tetramerization Domain Containing 18; involved in cell proliferation, differentiation, apoptosis, and metabolism.	UUUCUUACUCUUUUCUGUUA
ath-miR156g	NM_001256272|*VSX1*	2.5	Visual System Homeobox 1; involved in the regulation of cone opsin genes during early development.	UUUUUCACUUUCUUCUGUUG
ath-miR156g	NM_005737|*ARL4C*	2.5	ADP-ribosylation factor-like protein 4C; involved in vesicular trafficking and cell proliferation.	UUUUUCACUUUCUUCUGUUG
ath-miR156i	NM_014873|*LPGAT1*	2.0	Lysophosphatidylglycerol Acyltransferase 1; involved in lipid metabolism.	AUGUUCUCUCUCUUCUGUUU
ath-miR156i	NM_001128933|*SYNPO2*	2.5	Synaptopodin-2 a (Myopodin), actin-binding protein; regulates actin cytoskeleton through the RhoA signaling pathways.	CUGCUCUUUUUCUGCUGUCA
ath-miR156i	NM_001031848|*SERPINB8*	2.5	Serpin B8: member of the serine protease inhibitor (serpin) family; regulates inflammation and immune responses.	AUGCUCUUUGUCUUUUGUCA
ath-miR156i	NM_006241|*PPP1R2*	2.5	Protein Phosphatase 1 Regulatory Inhibitor Subunit 2; involved in cell division, muscle contraction, and neuronal activities.	UUUCUCUCACUCUUCUGUCA
ath-miR156i	NM_207645|*C11orf87*	2.5	Neuronal Integral Membrane Protein 1.	UUGUAUUCUCUUUUCUGUCA
ath-miR156i	NM_014048|*MKL2*	2.5	Myocardin-Related Transcription Factor B, transcriptional coactivator of SRF; regulates the expression of genes involved in myogenic differentiation and motility.	UGGCCCUCUUUCUUCUGUCA
ath-miR156i	NM_001199876|*SERF2*	2.5	Small EDRK-Rich Factor 2; involved in cell aggregation and stress responses.	GUUCACUUUCUCUUCUGUCA
ath-miR319c	NM_014906|*PPM1E*	2.0	Protein Phosphatase, Mg^2+^/Mn^2+^ Dependent 1E, inhibitor of kinase activity; affects cytoskeletal remodeling and metabolic regulation.	AAGUAGUUCCCUUCAGUUCAA
ath-miR168a-5p	NM_145166|*ZBTB47*	2	Putative DNA-binding transcription factor; involved in the regulation of transcription.	UCCCUGACCUGCACCAGGUGG
ath-miR164b-5p	NM_022492|*TTC31*	2.5	Tetratricopeptide Repeat Domain 31; involved in Adams-Oliver Syndrome (skin and limb abnormalities).	AGCUCGUGCCCUGUUUUUCUA
ath-miR164b-5p	NM_014982|*PCNX*	2.5	Pecanex Homolog (PCNX1); involved in cellular signaling.	CCCAUGUGCUCUGCUUCUCUC
ath-miR164b-5p	NM_016257|*HPCAL4*	2.5	Hippocalcin-Like Protein 4; involved in neuronal calcium signaling.	CCCACCUGUCCUGCUUCUCUG

## Data Availability

Proteomic data have been deposited with the ProteomeXchange Consortium via the PRIDE partner repository [57], with the dataset identifier PXD068478 (https://www.ebi.ac.uk/pride/archive (accessed on 16 November 2025)). Lipidomic data have been deposited at the MetaboLights database [58] with the dataset identifier MTBLS13341 (https://www.ebi.ac.uk/metabolights/ (accessed on 16 November 2025)). MicroRNA sequence data were deposited at the SRA database with the dataset identifier PRJNA1306069 (https://www.ncbi.nlm.nih.gov/sra/PRJNA1306069 (accessed on 16 November 2025)).

## Supplementary Materials

The supporting information can be downloaded at: https://www.mdpi.com/article/10.3390/ijms262311261/s1.

## Abbreviations

The following abbreviations are used in this manuscript:

PDNVs	Plant-Derived Nanovesicles
MDEs	Mammal-Derived Exosomes
HEs	Human Exosomes
TEM	Transmission Electron Microscopy
NTA	Nanoparticle Tracker Analysis
